# Effects of Vitamin E Supplementation on Growth Performance, Antioxidant Capacity, and Hepatic Lipid Metabolism in Juvenile Chinese Sturgeon (*Acipenser sinensis*)

**DOI:** 10.3390/antiox14111347

**Published:** 2025-11-10

**Authors:** Pei Chen, Wei Jiang, Xu Cheng, Baifu Guo, Yacheng Hu, Xiaofang Liang, Kaiyong Jiang, Wenchao Zhu, Jing Yang

**Affiliations:** 1 Hubei Key Laboratory of Three Gorges Projects for Fishes Conservation, Chinese Sturgeon Research Institute of China Three Gorges Corporation, Yichang 443100, China; chen_pei1@ctg.com.cn (P.C.); jiang_wei6@ctg.com.cn (W.J.); cheng_xu2@ctg.com.cn (X.C.);; 2 National Aquafeed Safety Assessment Center, Institute of Feed Research, Chinese Academy of Agricultural Sciences, Beijing 100081, China; 3 The Office of Yangtze River Basin Fishery Supervision and Administration, Ministry of Agriculture and Rural Affairs, Shanghai 200333, China

**Keywords:** Chinese sturgeon, vitamin E, growth performance, antioxidant capacity, lipid metabolism

## Abstract

This study investigated the effects of vitamin E supplementation on growth performance, antioxidant capacity, and hepatic lipid metabolism in one-year-old juvenile Chinese sturgeon (*Acipenser sinensis*). A total of 270 fish (initial weight 1.37 ± 0.04 kg) were allocated into 9 fiberglass tanks and fed isonitrogenous and isolipidic diets with graded concentrations of vitamin E (DL-α-tocopherol acetate) including, 0, 1000, and 2000 mg/kg, respectively, for 2 months. Results showed that 1000 mg/kg vitamin E significantly improved growth performance and decreased hepatosomatic index. Dietary vitamin E supplementation significantly reduced the hepatic crude protein and crude lipid levels, withnot significantly affecting moisture and crude ash. Dietary vitamin E led to significant increases in plasma high-density lipoprotein cholesterol and vitamin E levels, while decreasing plasma low-density lipoprotein cholesterol. Additionally, it raised liver vitamin E content and reduced hepatic triglycerides, total cholesterol, crude protein, and crude lipid levels. Vitamin E also significantly downregulated mRNA levels of lipogenesis-related genes (*ACC1*, acetyl-CoA carboxylase 1; *FASN*, fatty acid synthase; and *PPARγ*, peroxisome proliferator activated receptor γ) and inhibited the enzyme activities of ACC1 and FASN, while upregulating lipolysis-related genes (*HSL*, hormone-sensitive lipase; *CPT1*, carnitine palmitoyltransferase 1*α*, and *PPARα*, peroxisome proliferator activated receptor *α*) and enhancing the activities of HSL and CPT1α. Furthermore, vitamin E supplementation significantly improved plasma reduced glutathione and superoxide dismutase activities, lowered plasma reactive oxygen species, malondialdehyde levels, and hepatic malondialdehyde contents, and upregulated mRNA levels of hepatic *Nrf2* (nuclear factor-erythroid 2 related factor 2), *Keap1* (Kelch-like epichlorohydrin associating protein 1), and *CuZnSOD* (copper/zinc superoxide dismutase). In conclusion, dietary 1000 mg/kg vitamin E supplementation could improve growth performance, enhance antioxidant capacity, and reduce liver fat deposition, indicating its potential as a beneficial dietary additive for promoting health and lipid regulation in juvenile Chinese sturgeon.

## 1. Introduction

Vitamin E, comprising eight fat-soluble tocopherols and tocotrienols, primarily functions as a lipid-soluble antioxidant [1]. Fish are unable to synthesize all forms of biologically active vitamin E and must acquire it through their diets [2]. Adequate dietary vitamin E is crucial for promoting growth, scavenging free radicals, preventing lipid peroxidation in cellular membranes, and mitigating oxidative stress in fish [3]. Nonetheless, the vitamin E requirements vary among different farmed fish species, ranging from 6 to 451 mg kg^−1^ of α-tocopherol [3,4,5]. In addition to its protective functions, vitamin E also modulates lipid metabolism by influencing fatty acid composition and associated metabolic pathways [6]. Vitamin E has been demonstrated to influence lipid accumulation by modulating the expression of genes implicated in lipid metabolism, including those associated with lipogenesis and lipolysis in species such as zebrafish (*Danio rerio*) [7], hybrid grouper (♀*Epinephelus fuscoguttatus* × ♂*Epinephelus lanceolatus*) [8], and golden pompano (*Trachinotus ovatus*) [9]. However, the current understanding of vitamin E’s role in the lipid metabolic processes of fish and other aquatic organisms remains scarce.

To date, several studies related to vitamin E in sturgeons were reported in Cobice sturgeon (*Acipenser naccarii*) [10], lake sturgeon (*Acipenser fulvescens* R.) [11], beluga sturgeon (*Huso huso* L.) [12], and stellate sturgeon (*Acipenser stellatus*) [13]. Sub-yearling beluga sturgeon fed diets with 20–250 mg kg^−1^ of DL-all-rac-α-tocopherol acetate exhibited significantly increased final body weight, with no notable impacts on muscle composition or hematological and immunological parameters [12]. Nevertheless, information on the requirement of vitamin E and its effects on the lipid metabolic pathways in sturgeon remains insufficiently explored [14].

Chinese sturgeon (*Acipenser sinensis*), a significant anadromous fish native to the Yangtze River, has evolved over 200 million years and holds great ecological and cultural value [15]. Unfortunately, this species faces imminent extinction due to habitat fragmentation, overexploitation, and anthropogenic pressures [16]. Currently, the Chinese sturgeon depends significantly on artificial breeding and release programs for population sustainability [17]. While effective captive breeding is vital for its conservation, optimizing nutritional strategies to enhance juvenile growth, immune response, and stress resilience poses ongoing challenges. Current conservation efforts for the Chinese sturgeon focus on captive breeding; however, inadequate nutrition in artificial diets may impede the survival and growth of juvenile sturgeons [18]. These juveniles grow rapidly, requiring strong antioxidant defenses to manage oxidative stress from their metabolic activities.

Therefore, the current investigation employed a comprehensive array of physiological, biochemical, and molecular methodologies to assess the impact of dietary vitamin E supplementation on the growth performance, antioxidant status, and lipid metabolism in juvenile Chinese sturgeon. This study aims to elucidate the role of vitamin E in enhancing the health and viability of captive Chinese sturgeons, thereby offering essential insights for the formulation of nutritionally optimized diets.

## 2. Material and Methods

Throughout the experimental duration, the fish were handled in accordance with the Laboratory Animal Welfare Guidelines of the Hubei Key Laboratory of Three Gorges Projects for Fish Conservation.

### 2.1. Experimental Diets

Three isonitrogenous and isolipidic diets were formulated for this study, containing 0 (V0), 1000 (V1), and 2000 (V2) mg/kg vitamin E. The protein sources included fish meal, chicken protein meal, and cottonseed protein concentrate, while fish oil, soy lecithin, and lecithin oil served as the lipid sources. Vitamin E was incorporated in the form of DL-α-tocopherol acetate, with 50% purity, procured from Jilin Beisha Pharmaceutical Co., Ltd. The dry components were finely ground, thoroughly mixed, and extruded into 3 mm diameter sinking pellets using a twin-screw extruder (EXT50A, Yanggong Machine, Yangzhou, Jiangshu, China). All diets underwent air drying at ambient temperature and were subsequently stored at −20 °C until required. The formulations and nutrient profiles of the diets are detailed in Table 1.

### 2.2. Feeding Trial

One-year-old juvenile Chinese sturgeon were obtained from the Chinese Sturgeon Research Institute (Yichang, China) and housed in cylindrical fiberglass tanks. Prior to the trial, the fish experienced a one-week acclimatization phase, during which they were fed the basal diet (V0) at a daily rate of 3% of their body weight (BW/d). A fasting period of 24 h preceded the commencement of the feeding trial.

A total of 270 fish (initial average weight of 1.37 ± 0.04 kg) were randomly allocated into nine cylindrical fiberglass tanks (3 m in diameter and 1.5 m in height), with 30 fish per tank. Each tank was supplied with a continuous flow of water (approximately 12 L per min), maintaining a depth of 0.8 m, and was randomly assigned to one of three replicates of the three diets. The fish were fed twice daily (9:00 and 16:00 h) at a rate of 3% BW/d over a period of 2 months. Approximately 200% of the water in each tank was replaced daily, and siphoning was employed to remove fecal matter. During the feeding trial, water temperature was regulated at 18.2 ± 1.2 °C, with pH levels between 7.1 and 7.4, dissolved oxygen concentrations exceeding 7.5 mg/L, and ammonia-N levels below 0.1 mg/L. The fish were maintained under a natural light and dark cycle.

### 2.3. Sample Collection

At the conclusion of the feeding trial, all fish were subjected to a 24 h fasting period and anesthetized with MS-222 (200 mg/L). The fish were then counted and weighed for the evaluation of specific growth rate (SGR), weight gain rate (WGR), survival rate (SR), feeding rate (FR), and feed conversion ratio (FCR). Five fish from each tank were randomly selected for measurement of body weight and liver weight to facilitate the calculation of hepatosomatic index (HSI). Then, blood samples from 4 fish per tank were swiftly collected from the caudal vein, centrifuged (4000× *g* for 10 min) at 4 °C to obtain plasma for the analysis of hematological parameters. Liver samples from 4 fish per tank were dissected, immediately frozen in liquid nitrogen, and stored at −80 °C for the subsequent analyses of tissue homogenate, mRNA isolation, and nutrient composition.

### 2.4. Nutrient Composition Analysis

The chemical compositions of the feed and liver samples were analyzed in accordance with the standard methodologies established by the Association of Official Analytical Chemists [19]. Moisture content was assessed by drying samples at 105 °C until reaching a constant weight. Crude protein content was determined via the Kjeldahl method, crude lipid through Soxhlet extraction using diethyl ether, and ash content by incineration in a muffle furnace at 550 °C for 4 h.

### 2.5. Plasma and Liver Homogenate Parameters

Plasma glucose (GLU), urine nitrogen (BUN), triglycerides (TG), total cholesterol (TC), high-density lipoprotein cholesterol (HDL-C), low-density lipoprotein cholesterol (LDL-C), vitamin E, total antioxidant capacity (T-AOC), reduced glutathione (GSH), catalase (CAT), superoxide dismutase (SOD), malondialdehyde (MDA), along with liver TG, TC, vitamin E, T-AOC, GSH, CAT, SOD, and MDA were quantified using commercial assay kits (Nanjing Jiancheng Co., Ltd., Nanjing, Jiangshu, China) as per the provided protocols. Furthermore, plasma reactive oxygen species (ROS), liver ROS, acetyl-CoA carboxylase 1 (ACC1), fatty acid synthase (FASN), adipose triglyceride lipase (ATGL), hormone-sensitive lipase (HSL), and carnitine palmitoyl transferase 1 (CPT1α) were measured using commercial ELISA assay kits for fish (Keep Biotechnology Co., Ltd., Wuhan, Hubei, China) following the prescribed procedures. All physiological parameters were assessed with a microplate reader (PowerWave XS2, BioTek, Minneapolis, MN, USA) according to the guidelines.

### 2.6. Quantitative PCR (qPCR) Analysis

Total RNA from the liver was extracted using TRNzol Universal total RNA extraction reagent (TianGen, Beijing, China) in line with the manufacturer’s instructions. The extracted RNA was quantified with an Ultramicro spectrophotometer NanoDrop 2000c (Thermo Sci, Waltham, MA, USA), and its quality was evaluated through electrophoresis on a 1.5% (*w*/*v*) agarose gel. First-strand cDNA was synthesized from 1.5 μg of the total RNA using the TUREscript RT MasterMix (OneStep gDNA Removal) (Keep Biotechnology, China), which served as the template. The 2 x Dual Sybr Green qPCR Mix (Universal ROX) (Keep Biotechnology, China) was utilized for qPCR detection on the AB Step One Plus Real-Time PCR system (Applied Biosystems, Foster City, CA, USA). Each 20 μL reaction comprised 10 μL of 2x SYBR, 2 μL of cDNA template, 0.5 μL of each 10 μM specific primer, and 7 μL of RNA-free water. The amplification conditions were set at 95 °C for 2 min for pre-denaturation, followed by 15 s of denaturation at 95 °C, and 34 s of annealing and extension at 60 °C, for a total of 35 cycles. Each sample underwent triplicate runs, and data were analyzed using the Pfaffl method with two reference genes [20]. The gene-specific primers for mRNA quantification via qPCR are listed in Table 2. Serial dilutions of cDNA derived from liver tissues were employed to create a standard curve to assess the amplification efficiency of target and reference genes. Elongation factor 1α (*EF1α*) and glyceraldehyde-3-phosphate dehydrogenase (*GAPDH*) were used as endogenous reference genes [21].

### 2.7. Statistical Analysis

Statistical analyses were performed with SPSS software version 26.0. Variance homogeneity was evaluated before data analysis. A one-way analysis of variance (ANOVA) followed by Duncan’s post hoc test was conducted to identify significant differences (*p* < 0.05) among treatment groups. Results are presented as mean ± standard error, with graphical representations created using GraphPad Prism Software version 9.0.

## 3. Results

### 3.1. Growth Performance, Morphometric Parameters, and Liver Nutrient Compositions

The effects of dietary vitamin E on the growth performance and morphometric parameters of juvenile Chinese sturgeon were shown in Table 3. The SR for all experimental groups was 100%. The FBW, WGR, and SGR in the V1 group was significantly higher than that in the V0 and V2 groups (*p* < 0.05). The HSI in the V1 group was significantly lower than that in the V0 group (*p* < 0.05). However, no significant differences were observed in the FCR and FR among the groups (*p* > 0.05).

The liver nutrient compositions were presented in Table 4. The levels of crude protein and crude lipid in the liver were significantly reduced in the V1 and V2 groups compared to the V0 group (*p* < 0.05), while moisture and crude ash did not show significant variations (*p* > 0.05).

### 3.2. Antioxidant Capacity and Vitamin E Contents

**Figure 1:**
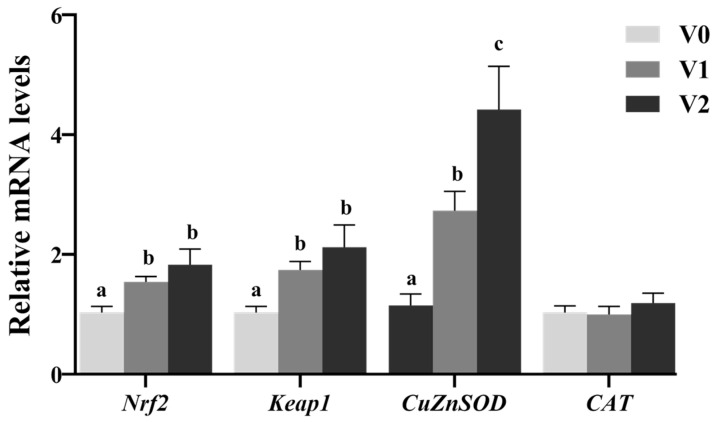
Relative mRNA levels of hepatic antioxidant-related genes of juvenile Chinese sturgeon fed diets with different vitamin E levels. V0, 0 mg/kg vitamin E; V1, 1000 mg/kg vitamin E; V2, 2000 mg/kg vitamin E. For the full name of the genes, refer to Table 2. The different letters above bar graphs indicate significant difference (*p* < 0.05).

The effects of vitamin E on plasma and liver antioxidant parameters in juvenile Chinese sturgeon were presented in Table 5. Compared to the V0 group, both plasma ROS and MDA levels significantly decreased in the V1 and V2 groups (*p* < 0.05). Plasma GSH activity significantly increased with elevated dietary vitamin E (*p* < 0.05), while CAT activity significantly rose only in the V2 group (*p* < 0.05). Plasma SOD activity significantly increased in the V1 group compared to the V0 group (*p* < 0.05) and subsequently decreased in the V2 group (*p* < 0.05). No significant differences were noted in plasma T-AOC (*p* > 0.05). In the liver, ROS, T-AOC, GSH, CAT, and SOD activities showed no significant changes with varying dietary vitamin E levels (*p* > 0.05). However, hepatic MDA level significantly declined in the V1 and V2 groups relative to the V0 group (*p* < 0.05). The mRNA levels of *Nrf2* and *Keap1* in the liver were significantly upregulated in the V1 and V2 groups compared to the V0 group (*p* < 0.05). The mRNA level of *CuZnSOD* was significantly upregulated with higher dietary vitamin E levels (*p* < 0.05), while no significant changes were observed in *CAT* mRNA levels (*p* > 0.05) (Figure 1).

**Figure 2:**
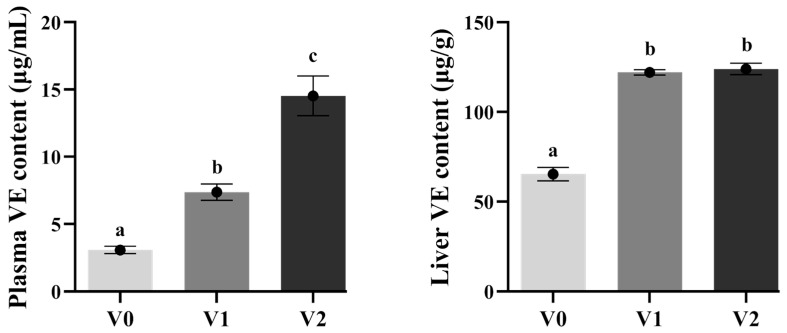
Vitamin E levels in plasma and liver of juvenile Chinese sturgeon fed with different vitamin E diets. V0, 0 mg/kg vitamin E; V1, 1000 mg/kg vitamin E; V2, 2000 mg/kg vitamin E. The different letters above bar graphs indicate significant difference (*p* < 0.05).

The plasma vitamin E levels significantly increased with higher dietary vitamin E levels (*p* < 0.05), and the hepatic vitamin E content also significantly improved (*p* < 0.05) before stabilizing (Figure 2).

### 3.3. Biochemical Parameters

The effects of vitamin E on plasma and liver biochemical parameters in juvenile Chinese sturgeon were shown in Table 6. There were no significant differences in plasma GLU and BUN levels across the groups (*p* > 0.05). However, plasma TG and TC levels in the V1 group were significantly higher compared to the V2 group (*p* < 0.05), with no significant differences observed in the V0 group (*p* > 0.05). The plasma HDL-C level in the V1 and V2 groups were significantly elevated compared to the V0 group (*p* < 0.05), while LDL-C level decreased as dietary vitamin E levels increased (*p* < 0.05). Additionally, hepatic TG and TC levels in the V1 and V2 groups were significantly lower than in the V0 group (*p* < 0.05).

### 3.4. Hepatic Lipid Metabolism

**Figure 3:**
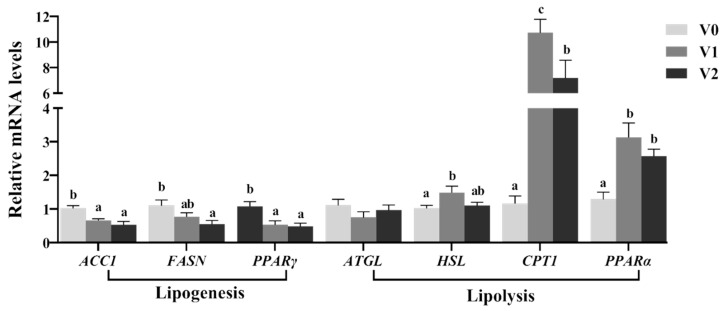
Relative mRNA levels of hepatic lipid metabolism-related genes in juvenile Chinese sturgeon fed diets with different vitamin E levels. V0, 0 mg/kg vitamin E; V1, 1000 mg/kg vitamin E; V2, 2000 mg/kg vitamin E. For the full name of the genes, refer to Table 2. The different letters above bar graphs indicate significant difference (*p* < 0.05).

The mRNA levels and enzymatic activities of genes related to hepatic lipid metabolism were presented in Figure 3 and Table 7. In the V1 and V2 groups, the mRNA levels of genes involved in hepatic lipogenesis (*ACC1*, *FASN*, and *PPARγ*) were significantly downregulated compared to the V0 group (*p* < 0.05). Conversely, the mRNA levels of genes associated with TG hydrolysis (*HSL*) and β-oxidation (*PPARα* and *CPT1*) were significantly upregulated (*p* < 0.05), while no significant differences were noted in *ATGL* mRNA levels (*p* > 0.05). Reflecting the mRNA expression patterns, the enzyme activities of ACC1 and FASN were significantly lower in the V1 and V2 groups (*p* < 0.05), whereas HSL and CPT1 activities showed significant increases (*p* < 0.05), with no significant difference noted for ATGL (*p* > 0.05).

## 4. Discussion

Vitamin E is integral to various physiological functions and is vital for the health and growth of fish [2]. In the present study, the V1 group (1000 mg/kg vitamin E) induced significant enhancements in FBW, WGR, and SGR. These findings aligned with previous studies on gilthead seabream (*Sparus aurata*) [22,23] and meagre (*Argyrosomus regius*) [24]. It is suggested that Chinese sturgeon may have a higher vitamin E requirement compared to other fish species due to their lipid and n-3 HUFA-rich diets [3,6]. Thus, sufficient dietary vitamin E is essential for reducing lipid oxidation and enhancing survival and growth, as demonstrated in sea bass (*Dicentrarchus labrax*) [25].

In contrast, the V2 group (2000 mg/kg vitamin E) exhibited a significant reduction in growth performance when compared to the V1 group, indicating that excessive vitamin E supplementation could hinder the growth of Chinese sturgeon. Similar findings had been reported in spotted snakehead (*Channa punctatus*) [26], grass carp [27], sea cucumber (*Apostichopus japonicus*) [28], and yellow catfish (*Pelteobagrus fulvidraco*) [29]. This result may be attributed to that elevated dietary vitamin E could induce lipid peroxidation, functioning as a prooxidant in fish species [1,3]. Consequently, the appropriate dietary vitamin E concentration for juvenile Chinese sturgeon seemed to be approximately 1000 mg kg^−1^.

Fish are unable to synthesize all biologically active forms of vitamin E and depend on external dietary sources for their acquisition [2]. The liver serves as the primary organ for vitamin E storage in fish. Consequently, within a certain range, the concentration of hepatic vitamin E significantly increases with higher dietary levels of vitamin E. In this study, liver vitamin E accumulation was notably elevated in the V1 and V2 groups, consistent with findings from other fish species such as sea bream (*Pagrus major*) [30], golden pompano [9], hybrid grouper [8], and Caspian trout [5]. Therefore, it was inferred that the enhanced vitamin E concentrations in the liver might be anticipated to bolster the hepatic antioxidant capacity in juvenile Chinese sturgeon.

As an antioxidant, vitamin E safeguards cell membranes from oxidative damage caused by ROS [3,6]. Under normal circumstances, the production and elimination of ROS are in balance. However, when environmental stress occurs, ROS levels can surge, exceeding the antioxidant system’s capacity to remove them, potentially leading to oxidative damage [31]. MDA, a marker for lipid peroxidation and lipid peroxides, can also indicate membrane damage inflicted by free radicals [32]. T-AOC, SOD, GSH, and CAT protecte cells from oxidative stress and the attendant damage by scavenging hydrogen peroxide, lipid peroxides, and the superoxide radical for organisms under stress [3,33,34]. Appropriate dietary vitamin E levels significantly decreased the content of MDA and increased activities of GSH and SOD in yellow catfish [29], largemouth bass (*Micropterus salmoides*) [35] and golden pompano [9]. These observations were consistent with the current study, where both the V1 and V2 groups exhibited significantly elevated plasma GSH and SOD activities, reduced ROS and MDA concentrations, along with higher vitamin E levels. These results suggested that vitamin E supplementation might enhance the antioxidant capacity of juvenile Chinese sturgeon.

The Nrf2-Keap1 signaling pathway was pivotal in the antioxidant defense system against oxidative stress [36]. Under oxidative stress, Nrf2 is liberated from Keap1 and moves into the nucleus, activating transcription, and also combines with the downstream antioxidant responsive element to activate antioxidant enzyme gene expression [37,38]. Previous studies had established that the activation of Nrf2 could modulate the mRNA levels of antioxidant enzyme genes, including *CuZnSOD*, *MnSOD*, and *CAT* [39,40,41]. Therefore, vitamin E has the capacity to directly induce Nrf2 expression and facilitate its translocation to the nucleus [42,43]. Vitamin E positively influenced the mRNA levels of antioxidant-related genes by upregulating *Nrf2* mRNA levels in grass carp (*Ctenopharyngodon idella*) [44], northern whiting (*Sillago sihama*) [41], and hybrid grouper [8]. Consistent with these findings, the current study demonstrated that vitamin E significantly upregulated the mRNA levels of *Nrf2*, *Keap1*, and *CuZnSOD* in the liver of juvenile Chinese sturgeon. These outcomes further suggested that vitamin E functions not only as a non-enzymatic antioxidant but also enhances the enzymatic antioxidant defense mechanisms by modulating the Nrf2-Keap1 signaling pathway and antioxidant gene expression to augment antioxidant capacity. Therefore, dietary vitamin E supplementation is a potential protective strategy against future oxidative challenges.

Beyond its roles in promoting growth and scavenging ROS, vitamin E also influences lipid metabolism [6]. Plasma lipid constituents (LDL and HDL) serve as critical biochemical markers indicative of the organism’s health status [45]. LDL-C is identified as a lipid metabolism disease factor owing to its involvement in atherosclerotic plaque formation, whereas HDL-C is recognized for its positive effects through the facilitation of reverse cholesterol transport from peripheral tissues to the liver for excretion [46,47]. In the present investigation, the marked increase in plasma HDL-C levels and decrease in LDL-C levels observed in the V1 and V2 groups implied that vitamin E might enhance lipid profiles and metabolic processes in juvenile Chinese sturgeon.

Lipid metabolism regulation encompasses both lipogenesis and lipolysis, which rely on various essential enzyme activities and transcription factors [48]. Research had demonstrated that vitamin E modulated lipid metabolism by influencing the mRNA levels of genes related to lipogenesis and lipolysis in fish [8,9,49]. ACC1 and FASN are pivotal enzymes in the de novo fatty acid synthesis pathway, their downregulation can result in diminished lipogenesis and hepatic lipid storage [50]. PPARγ, a transcription factor, plays a significant role in lipid metabolism, where its downregulation is associated with decreased lipogenesis and increased lipolysis [51]. In this study, the mRNA levels of hepatic lipogenesis-related genes (*ACC1*, *FASN*, and *PPARγ*) along with the enzymatic activities of ACC1 and FASN were markedly downregulated in the V1 and V2 groups, indicating that vitamin E might inhibit hepatic lipogenesis in juvenile Chinese sturgeon. Conversely, the mRNA levels of genes associated with triglyceride hydrolysis and β-oxidation (*HSL*, *PPARα*, and *CPT1*) and the enzymatic activities of HSL and CPT1 were significantly elevated. HSL, a crucial enzyme for triglyceride hydrolysis in adipose tissue and the liver, when upregulated can promote lipolysis and mitigate fat accumulation; similarly, CPT1, a rate-limiting enzyme in fatty acid β-oxidation, when upregulated facilitates the transfer of fatty acids into mitochondria for oxidation [50,52]. PPARα, a transcription factor that governs the expression of genes involved in fatty acid β-oxidation, when upregulated can enhance lipid metabolism and energy production [52]. The notable upregulation of these genes in the V1 and V2 groups further supports the hypothesis that Vitamin E may augment lipolysis and β-oxidation, suggesting a reduction in hepatic lipid accumulation and an improvement in lipid metabolism for juvenile Chinese sturgeon. Liang et al. [8] reported that diets containing 98 to 193 mg/kg of vitamin E significantly reduced FASN activity and mRNA levels while enhancing HSL activity and mRNA levels in the liver of hybrid grouper, consistent with our findings.

The liver serves as a crucial reservoir of energy reserves, making the HSI a useful indicator of the nutritional and energy status in fish [53]. Amlashi et al. [12] found that a deficiency in dietary vitamin E resulted in elevated HSI and increased hepatic lipid accumulation in Caspian trout. Li et al. [27] reported that vitamin E supplementation enhanced growth performance in grass carp through improved feed efficiency and reduced HSI. Vitamin E has been shown to significantly lower whole-body lipid content and HSI value in red sea bream [30] and blunt snout bream (*Megalobrama amblycephala*) [49]. In line with these observations, the present study found substantial reductions in HSI, hepatic crude lipid, TG, and TC levels in the V1 group, suggesting that a dietary inclusion of 1000 mg/kg vitamin E may facilitate lipid metabolism and reduce hepatic lipid accumulation, thereby improving health and enhancing the growth performance of Chinese sturgeon [47]. Unfortunately, the study did not include a longer growth period to investigate the interplay between hepatic metabolism and histopathology in Chinese sturgeon.

## 5. Conclusions

The current study demonstrated that dietary 1000 mg/kg vitamin E supplementation could significantly improve growth performance, enhance antioxidant capacity, and reduce liver lipid deposition in juvenile Chinese sturgeon. The mechanisms underlying these effects include the reduction in oxidative stress, the upregulation of antioxidant genes, the inhibition of lipogenesis, and the enhancement of lipolysis and β-oxidation. These findings provide valuable insights into the role of vitamin E in promoting health and lipid regulation in juvenile Chinese sturgeon, and suggest that vitamin E can be a beneficial dietary additive for enhancing the growth and health of this species in captive breeding programs. Further research is needed to optimize the dietary vitamin E levels and to explore the long-term interplay between hepatic metabolism and histopathology in Chinese sturgeon.

## Figures and Tables

**Table 1:** Formulation and nutrient compositions of experimental diets.

Ingredients (g/kg)	V0	V1	V2
Fish meal	400	400	400
Chicken powder meal	150	150	150
Cottonseed protein concentrate	150	150	150
Wheat flour	180	180	180
Fish oil	40	40	40
Soybean oil	30	30	30
Lecithin oil	15	15	15
Vitamin and mineral premix ^a^	12	12	12
Ca(H_2_PO_4_)_2_	8	8	8
Choline chloride (60%)	4	4	4
Bentonite	11	9	7
Vitamin E (50%)		2	4
Total	1000	1000	1000
*Analyzed chemical composition (dry matter basis, %)*			
Moisture	6.45	6.67	6.56
Crude ash	11.65	11.42	11.46
Crude protein	50.21	49.97	50.11
Crude lipid	13.35	13.63	13.45

V0, 0 mg/kg vitamin E; V1, 1000 mg/kg vitamin E; V2, 2000 mg/kg vitamin E. ^a^ Vitamin premix (mg/kg diet): Vitamin A 20; Vitamin D_3_ 10; Vitamin K_3_ 20; Vitamin B_1_ 10; Vitamin B_2_ 15; Vitamin B_6_ 15; Vitamin B_12_ (1%) 8; Ascorbic acid (35%) 1000; Calcium pantothenate 40; Niacinaminde 100; Inositol 200; Biotin (2%) 2; Folic acid 10; Corn gluten meal 550. Mineral premix (mg/kg diet): CuSO_4_·5H_2_O 10; FeSO_4_·H_2_O 300; ZnSO_4_·H_2_O 200; MnSO_4_·H_2_O 100; KI (10%) 80; CoCl_2_·6H_2_O (10%Co) 5; Na_2_SeO_3_ (10% Se) 10; MgSO_4_·5H_2_O 2000; NaCl 2100; Zeolite 4995; Antioxidant 200.

**Table 2:** Primer sequences used for gene expression quantification by qPCR in this study.

Gene Names	Forward Primer (5′-3′)	Reverse Primer (5′-3′)
*EF1α*	TGGCATCACCATTGACATCT	AGCTGCTTCACACCCAGAGT
*GAPDH*	CATTGCCAGGGAAACACTTT	ACACCGGGCTAGACAAACAC
*ACC1*	CCGCAACATGCTGTTTCTTA	GCACTGATGCCTTTTTGACA
*FASN*	AGGAGGTGGAAACCAGGACT	ATCCACAATGGCTTCGTAGG
*PPARγ*	AACCTGCTGGATTTGTTTGG	CAGCTGTGACATAGCCTGGA
*ATGL*	CTCTGGGTTGGGTGAGAAAA	TTTCATGCCCTACTGGGAAG
*HSL*	GTTCCTGGGACCTCCTTCTC	CCTCCGTGAGGAAGAAACTG
*CPT1α*	AGGGAGAGTTCTGCCTGACA	CAGAAAAGGTGCCGATCAAT
*PPARα*	CTGGAGCTGGATGACAGTGA	GGAGTAGCTTGGGGAAAAGG
*Nrf2*	TAACCATCCCCATGGTAGGA	TGGCTGAAGGTTTCGAGAGT
*Keap1*	GTGGGAGAGAAGTGCGTCAT	CTGGCAGTGAGACAGGTTGA
*CuZnSOD*	TTGTGTGAGTGCTGGTCCTC	CAGGCTCTCGTCATTTCCTC
*CAT*	GCAAGGGCAACTGTTTGAAT	ATACGGTCGGCTGTATGAGG

*EF1α*, elongation factor 1α; *GAPDH*, glyceraldehyde-3-phosphate dehydrogenase; *ACC1*, acetyl-CoA carboxylase 1; *FASN*, fatty acid synthase; *PPARγ*, peroxisome proliferator activated receptor γ; *ATGL*, adipose triglyceride lipase; *HSL*, hormone-sensitive lipase; *CPT1α*, carnitine palmitoyltransferase 1α; *PPARα*, peroxisome proliferator activated receptor α; *Nrf2*, nuclear factor-erythroid 2 (NF-E2)-related factor 2; *Keap1*, Kelch-like epichlorohydrin (ECH) associating protein 1; *CuZnSOD*, copper/zinc superoxide dismutase; *CAT*, catalase.

**Table 3:** Growth performance and morphometric parameters of juvenile Chinese sturgeon fed diets with different vitamin E levels.

	V0	V1	V2
IBW ^a^	1.36 ± 0.04	1.37 ± 0.03	1.36 ± 0.03
FBW ^b^	3.68 ± 0.13 ^a^	4.08 ± 0.11 ^b^	3.71 ± 0.11 ^a^
WGR ^c^	168.30 ± 11.15 ^a^	197.81 ± 12.06 ^b^	170.80 ± 10.31 ^a^
SGR ^d^	1.67 ± 0.05 ^a^	1.85 ± 0.06 ^b^	1.69 ± 0.06 ^a^
FCR ^e^	1.13 ± 0.03	1.11 ± 0.04	1.15 ± 0.04
FR ^f^	1.37 ± 0.04	1.41 ± 0.06	1.43 ± 0.07
HIS ^g^	3.34 + 0.09 ^b^	2.93 ± 0.13 ^a^	3.13 ± 0.13 ^ab^
SR ^h^	100	100	100

V0, 0 mg/kg vitamin E; V1, 1000 mg/kg vitamin E; V2, 2000 mg/kg vitamin E. Within the same row, values with different superscripts are significantly different (*p* < 0.05). ^a^ IBW(kg/fish): the initial body weight, n = 3. ^b^ FBW(kg/fish): the final body weight, n = 3. ^c^ WGR (weight gain rate, %) = 100 × (W_f_ − W_i_)/W_i_, n = 3. ^d^ SGR (specific growth rate, %) = 100 × [Ln (W_f_/W_i_)]/days, n = 3. ^e^ FCR (feed conversion ratio) = feed intake/(W_f_ − W_i_), n = 3. ^f^ FR (feeding rate, %) = 100 × feed intake/[(W_f_ + W_i_)/2]/days, n = 3. ^g^ HSI (hepatosomatic index, %) = 100 × liver weight/W_f_, n = 15. ^h^ SR (Survival rate, %) = 100 × final fish number/initial fish number, n = 3. W_i_ is the initial body weight, W_f_ is the final body weight.

**Table 4:** Liver nutrient compositions of juvenile Chinese sturgeon fed diets with different vitamin E levels (%, in dry matter basis).

	V0	V1	V2
Moisture	48.41 ± 0.80	47.63 ± 1.22	49.43 ± 1.26
Crude protein	18.48 ± 0.26 ^b^	14.58 ± 1.02 ^a^	15.87 ± 0.62 ^a^
Crude lipid	75.80 ± 0.67 ^b^	68.31 ± 1.38 ^a^	71.88 ± 0.94 ^a^
Crude ash	2.04 ± 0.25	2.40 ± 0.27	1.98 ± 0.27

V0, 0 mg/kg vitamin E; V1, 1000 mg/kg vitamin E; V2, 2000 mg/kg vitamin E. Within the same row, values with different superscripts are significantly different (*p* < 0.05).

**Table 5:** Plasma and liver antioxidant parameters of juvenile Chinese sturgeon fed diets with different vitamin E levels.

	V0	V1	V2
*Plasma parameter*			
ROS μg/L	480.04 ± 7.27 ^b^	445.06 ± 10.46 ^a^	432.65 ± 9.32 ^a^
T-AOC μmol/L	286.41 ± 16.66	275.36 ± 19.62	260.91 ± 18.39
GSH μmol/L	305.10 ± 19.66 ^a^	358.84 ± 18.61 ^b^	419.65 ± 14.53 ^c^
CAT U/mL	1.69 ± 0.20 ^a^	1.49 ± 0.07 ^a^	2.27 ± 0.23 ^b^
SOD U/L	15.65 ± 1.69 ^a^	30.02 ± 2.69 ^c^	22.86 ± 1.57 ^b^
MDA nmol/L	4.67 ± 0.27 ^b^	3.09 ± 0.20 ^a^	3.01 ± 0.32 ^a^
*Liver parameter*			
ROS ng/g prot	220.83 ± 12.18	227.37 ± 15.13	208.60 ± 15.51
T-AOC μmol/g prot	26.70 ± 2.28	28.24 ± 3.57	33.29 ± 4.91
GSH μmol/g prot	156.87 ± 4.61	165.41 ± 14.88	142.66 ± 7.73
CAT U/mg prot	35.22 ± 1.60	34.66 ± 2.45	35.68 ± 5.62
SOD U/g prot	35.03 ± 1.30	35.52 ± 1.21	33.31 ± 1.24
MDA mmol/g prot	7.46 ± 0.72 ^b^	5.40 ± 0.40 ^a^	4.27 ± 0.41 ^a^

V0, 0 mg/kg vitamin E; V1, 1000 mg/kg vitamin E; V2, 2000 mg/kg vitamin E; ROS, reactive oxygen species; T-AOC, total antioxidant capacity; GSH, reduced glutathione; CAT, catalase; SOD, superoxide dismutase; MDA, malondialdehyde. Within the same row, values with different superscripts are significantly different (*p* < 0.05).

**Table 6:** Plasma and liver biochemical parameters in juvenile Chinese sturgeon fed diets with different vitamin E levels.

	V0	V1	V2
*Plasma parameter*			
GLU mmol/L	11.22 ± 0.20	11.17 ± 0.60	10.85 ± 0.32
BUN mmol/L	1.91 ± 0.23	1.70 ± 0.19	2.14 ± 0.24
TG mmol/L	2.89 ± 0.15 ^ab^	3.13 ± 0.31 ^b^	2.50 ± 0.12 ^a^
TC mmol/L	1.55 ± 0.06 ^ab^	1.79 ± 0.08 ^b^	1.36 ± 0.17 ^a^
HDL-C mmol/L	177.74 ± 7.21 ^a^	244.38 ± 33.10 ^b^	255.86 ± 16.06 ^b^
LDL-C mmol/L	545.36 ± 37.41 ^c^	365.17 ± 23.67 ^b^	150.51 ± 15.56 ^a^
*Liver parameter*			
TG μmol/g prot	454.69 ± 52.10 ^b^	285.15 ± 25.29 ^a^	323.79 ± 38.74 ^a^
TC μmol/g prot	407.98 ± 21.31 ^b^	320.14 ± 17.14 ^a^	359.84 ± 20.80 ^a^

V0, 0 mg/kg vitamin E; V1, 1000 mg/kg vitamin E; V2, 2000 mg/kg vitamin E; GLU, plasma glucose; BUN, urine nitrogen; TG, triglycerides; TC, total cholesterol; HDL-C, high-density lipoprotein; cholesterol; LDL-C, low-density lipoprotein cholesterol. Within the same row, values with different superscripts are significantly different (*p* < 0.05).

**Table 7:** Enzymatic activities of hepatic lipid metabolism in juvenile Chinese sturgeon fed diets with different vitamin E levels.

	V0	V1	V2
ACC1 ng/g prot	216.17 ± 16.38 ^b^	175.32 ± 10.60 ^a^	159.44 ± 10.42 ^a^
FASN ng/g prot	13.90 ± 1.80 ^b^	9.90 ± 1.77 ^ab^	8.18 ± 0.92 ^a^
ATGL μg/g prot	2.01 ± 0.17	1.98 ± 0.17	2.24 ± 0.29
HSL μg/g prot	1.51 ± 0.08 ^a^	2.23 ± 0.39 ^a^	3.27 ± 0.22 ^b^
CPT1 ng/g prot	7.42 ± 0.56 ^a^	11.01 ± 0.72 ^b^	9.88 ± 0.81 ^b^

V0, 0 mg/kg vitamin E; V1, 1000 mg/kg vitamin E; V2, 2000 mg/kg vitamin E; ACC1, acetyl-CoA carboxylase 1; FASN, fatty acid synthase; ATGL, adipose triglyceride lipase; HSL, hormone-sensitive lipase; CPT1, carnitine palmitoyl transferase 1. Within the same row, values with different superscripts are significantly different (*p* < 0.05).

## Data Availability

The data that support the findings of this study are available from the corresponding author upon reasonable request.
